# Overexpression of peroxiredoxin I and thioredoxin1 in human breast carcinoma

**DOI:** 10.1186/1756-9966-28-93

**Published:** 2009-06-30

**Authors:** Mee-Kyung Cha, Kyung-Hoon Suh, Il-Han Kim

**Affiliations:** 1 Department of Biochemistry, Paichai University, Daejeon 302-735, Republic of Korea

## Abstract

**Background:**

Peroxiredoxins (Prxs) are a novel group of peroxidases containing high antioxidant efficiency. The mammalian Prx family has six distinct members (Prx I-VI) in various subcellular locations, including peroxisomes and mitochondria, places where oxidative stress is most evident. The function of Prx I in particular has been implicated in regulating cell proliferation, differentiation, and apoptosis. Since thioredoxin1 (Trx1) as an electron donor is functionally associated with Prx I, we investigated levels of expression of both Prx I and Trx1.

**Methods:**

We investigated levels of expression of both Prx I and Trx1 in breast cancer by real-time polymerase chain reaction (RT-PCR) and Western blot.

**Results:**

Levels of messenger RNA (mRNA) for both Prx I and Trx1 in normal human breast tissue were very low compared to other major human tissues, whereas their levels in breast cancer exceeded that in other solid cancers (colon, kidney, liver, lung, ovary, prostate, and thyroid). Among members of the Prx family (Prx I-VI) and Trx family (Trx1, Trx2), Prx I and Trx1 were preferentially induced in breast cancer. Moreover, the expression of each was associated with progress of breast cancer and correlated with each other. Western blot analysis of different and paired breast tissues revealed consistent and preferential expression of Prx I and Trx1 protein in breast cancer tissue.

**Conclusion:**

Prx I and Trx1 are overexpressed in human breast carcinoma and the expression levels are associated with tumor grade. The striking induction of Prx I and Trx1 in breast cancer may enable their use as breast cancer markers.

## Background

Organisms living under aerobic conditions are exposed to reactive oxygen species (ROS) such as superoxide anion (O_2_^-^), hydrogen peroxide (H_2_O_2_), and nitric oxide (NO), which are generated by redox metabolism, mainly in mitochondria. It has been demonstrated *in vitro *that ROS in small amounts participate in many physiological processes such as signal transduction, cell differentiation, apoptosis, and modulation of transcription factors [1-3]. All organisms, from prokaryotes to primates, are equipped with different defensive systems to combat the toxic processes of ROS. These defensive systems include antioxidant enzymes such as superoxide dismutases, catalases, glutathione peroxidases, and a new type of peroxidase, the rapidly growing family of peroxiredoxins (Prxs) [3,4].

The major functions of Prxs comprise cellular protection against oxidative stress, modulation of intracellular signaling through H_2_O_2 _as a second messenger molecule, and regulation of cell proliferation. Peroxiredoxins are capable of protecting cells from ROS toxicity and regulating signal transduction pathways that use c-Abl, caspases, nuclear factor-kappaB (NF-κB), and activator protein-1 to influence cell growth and apoptosis. Evidence is fast growing that oxidative stress is important not only for normal cell physiology but also for many pathological processes such as atherosclerosis, neurodegenerative diseases, and cancer [5-8]. Reactive oxygen species participate in carcinogenesis in all stages, including initiation, promotion, and progression [5] Levels of ROS such as O_2_^- ^are increased in breast cancer [9,10]. The production of ROS accelerates tumor induction [11]. In vitro, Prx genes I-IV are overexpressed when H_2_O_2 _concentration in cells is elevated [12].

Peroxiredoxin I, a cytosol form, is the most abundant and ubiquitously distributed member of the mammalian Prx family, and it has been identified in a large variety of organisms. It has been suggested that Prx I regulates cell proliferation and apoptosis by its interaction with oncogene products such as c-Abl. Peroxiredoxin I has been investigated in various human cancer samples as a potential marker. The reports cited above support that Prx I may be closely associated with cancers. Nevertheless, the connection between Prx I and cancer has not yet been clearly defined. Elevated expressions of Prx I have been observed in several human cancers, including lung, breast, esophagus, oral, and thyroid [13-15]. In oral squamous cell cancer, Yanagawa *et al*. [15] found low levels of Prx I expression associated with larger tumor masses, lymph node metastases, and poorly differentiated cancers. In contrast, Karihtala *et al*. [16] found no correlation between Prx I expression and clinicopathological features in breast cancer. Instead, levels of expression of Prxs III, IV, and V were significantly higher when breast cancers were poorly differentiated, suggesting their relationship to breast cancer.

There are two major Prx subfamilies. One subfamily uses two conserved cysteines (2-Cys), and the other uses one cysteine (1-Cys) to scavenge H_2_O_2 _and alkyl hydroperoxides. Four mammalian 2-Cys members (Prx I-IV) use thioredoxin (Trx) as the electron donor for antioxidation [17]. Thioredoxin as an antioxidant protein is induced by various kinds of oxidative stresses [18-21]. Similar to Prxs, Trx plays an important role in regulating cancer cell growth, for example, by modulating the DNA binding activity of transcription factors, including nuclear factor-κB, p53, and glucocorticoid and estrogen receptors [22-25]. Thioredoxin may be closely associated with cancers. Immunohistochemical analysis using anti-Trx antibody has shown the expression of Trx in a number of human cancer tissues, including liver, colon, pancreas, and uterine cervix [26-28]. These data indicate the possible involvement of Trx in the process of oncogenesis.

To investigate the significance of Prx I in breast cancers, we examined Prx I expression in 204 samples of breast cancer tissue, as a model tissue, using quantitative methods such as real time-polymerase chain reaction (RT-PCR) and Western blot, and we investigated association with cancer grade. Since Trx1 is functionally associated with Prx I as the electron donor, we also examined the expression of Trx in the same tissues. The association of Trx1 with Prx I may indicate a physiological role for Prx I in breast cancer.

## Methods

### Study Material for Real-Time PCR Analysis

We used Human Major 48 Tissues real-time (HMRT) quantitative PCR arrays, Cancer Survey real-time (CSRT 96-I) quantitative PCR arrays, and Human Breast Cancer real-time (BCRT I-V) qPCR arrays from OriGene (OriGene Technologies, Inc, Rockville, MD, USA). Simultaneous examination of the expression of target genes in 48 different tissues was performed using the HMRT array, which consisted of panels of first-strand complementary DNA (cDNA) from human tissues selected from individuals of different ethnicity. Expression levels of target genes in eight different cancers (breast, colon, kidney, liver, lung, ovary, prostate, and thyroid) were measured using the CSRT array, consisting of 12 samples from each cancer type with cancer stage from I to IV. Expression of target genes in breast cancer was examined using four different sets of arrays (BCRT I-IV) to test 192 samples and using the CSRT 96-I array to test 12 samples. In the 204 samples, grading was distributed as follows: stage 0 (normal), 19; stage I, 37; stage II, 76; stage III, 60; and stage IV, 12. The cancer tissue types consisted of ductal (*n *= 154), lobular (*n *= 13), metastatic (*n *= 12), and other histological types of cancer (*n *= 25), including medullary, mucinous, tubular, recurrent, and papillary. More clinicopathological information for each patient is described in OriGene's product sheet. TissueScan Cancer qPCR Arrays are panels of normalized cDNA prepared from pathologist-verified human tumor tissues. The cDNAs were prepared from high quality cancer tissues.

### Study Material for Immunological Analysis

Total membrane and soluble proteins from clinically defined human cancer and normal tissues were obtained from Capital Biosciences (Gaithersburg, MD, USA). The proteins were prepared from high quality and pathologist-verified cancer tissues The proteins from different individuals and matched paired individuals (normal tissue and primary cancers; primary and metastatic cancers) were used for immunological analysis. The clinical and pathological findings of the cancers are summarized in Table 1.

**Table 1:** Clinicopathological Features of Cancer Tissues Used in Immunological Study.

**Sample**	**Tissue**	**Appearance**	**Age/gender^1^**	**Clinical Diagnosis**
**BRN0**	Brain	Normal	26/M	Normal

**BRC0**	Brain	Tumor	40/M	Astrocytoma

**BEN0–4**	Breast	Normal	82/F. 45/F. 56/F. 64/F. 76/F	Normal

**BEC0**	Breast	Tumor	47/F	Medullary Carcinoma, Well Differentiated

**BEC1**	Breast	Tumor	40/F	Invasive Lobular Carcinoma

**BEC2**	Breast	Tumor	42/F	Adenocarcinoma, Moderately Differentiated

**BEC3**	Breast	Tumor	42/F	Fibroadenoma

**BEC4**	Breast	Tumor	50/F	Infiltrative Ductal Carcinoma

**CLN0**	Colon	Normal	60/F	Normal

**CLC0**	Colon	Tumor	48/M	Adenocarcinoma, Well Differentiated

**KDN0**	Kidney	Normal	83/F	Normal

**KDC0**	kidney	Tumor	43/F	Granular Cell Carcinoma

**LVN0**	Liver	Normal	30/M	Normal

**LVC0**	Liver	Tumor	65/M	Hepatic Cellular Carcinoma

**LUN0–4**	Lung	Normal	24/F. 26/M. 66/M. 71/M.76/F	Normal

**LUC0**	Lung	Tumor	72/M	Squamous Cell Carcinoma

**LUC1**	Lung	Tumor	33/M	Squamous Cell Carcinoma, Moderately Differenciated

**LUC2**	Lung	Tumor	51/F	Adenocarcinoma, Moderately Differentiated

**LUC3**	Lung	Tumor	58/M	Squamous Cell Carcinoma, Moderately Differenciated

**LUC4**	Lung	Tumor	61/M	Adenocarcinoma

**OVN0–4**	Ovary	Normal	74/F. 37/F. 62/F.69F. N/A/F	Normal

**OVC0**	Ovary	Tumor	51/F	Cystoadenocarcinoma

**OVC1**	Ovary	Tumor	42/F	Granular Cell Tumor

**OVC2**	Ovary	Tumor	51/F	Cystoadenoma

**OVC3**	Ovary	Tumor	57/F	Leiomyosarcoma, Well Differentiated

**OVC4**	Ovary	Tumor	Adult/F	Clear Cell Adenocarcinoma

**BE1N**	Breast	Adjacent Normal	70/F, same patient	Normal
		
**BE1P**	Breast	Primary Tumor		Invasive Ductal Carcinoma

**BE2P**	Breast	Primary Tumor	59/F, same patient	Breast Carcinoma
		
**BE2M**	Breast	Metastatic Tumor		Breast Tumor Metastasized to Lung

**CL1N**	Colon	Adjacent Normal	62/F. same patient	Normal
		
**CL1P**	Colon	Primary Tumor		Adenocarcinoma

**CL2P**	Colon	Primary Tumor	66/F. same patient	Adenocarcinoma
		
**CL2M**	Colon	Metastatic Tumor		Colon Tumor Metastasized to Lymph Node

**LU1N**	Lung	Adjacent Normal	46/M, same patient	Normal
		
**LU1P**	Lung	Primary Tumor		Squamous Cell Carcinoma

**LU2P**	Lung	Primary Tumor	75/M. same patient	Squamous Cell Carcinoma
		
**LU2M**	Lung	Metastatic Tumor		Lung Tumor Metastasized to Lymph Node

^1^The information of age & gender was placed in order (e.g., BEN0, 82/F; BEN1, 45/F; BEN2, 56/F; BEN2, 64/F; BEN3, 76/F). Zero series of sample (e.g., BRN0) were used in the experiment shown in Figure 7. Abbreviations: N, Normal; C, Cancer; P, Primary Cancer; M, Metastatic Cancer or Male; F, Female; N/A, not available; Br, Brain; BE, breast; LV, Liver; LU, Lung; OV, Ovary; CL, colon.

### Immunological Analysis

Immunoblotting was performed according to the manufacturer's instructions using the Amersham ECL Western blotting system (GE Healthcare, Chalfont St. Giles, United Kingdom). Anti-Prx I, anti-Prx II, anti-Trx1, and anti-copper/zinc (Cu/Zn) superoxide dismutase (SOD) rabbit polyclonal antibodies that have cross-reactivity with the corresponding human protein were purchased from AbFrontier (Seoul, Korea). Samples were fractionated by electrophoresis on a 4% to 20% gradient sodium dodecyl sulfate (SDS) polyacrylamide gel (PAGE) (GenScript Corp, Piscataway, NJ, USA) and transferred onto polyvinylidene difluoride membranes (Millipore, Billerica, MA, USA). The membranes were blocked and incubated with antibody (1:1000 by volume) in phosphate-buffered saline (PBS) containing 0.1% Tween 20 at room temperature for 2 hours. After extensive washing, the membranes were incubated with polyclonal goat anti-rabbit IgG antibody (1:2000 by volume) conjugated with horseradish peroxidase. The membranes were washed in PBS, and the chemiluminescent substrate was added. The membranes were stripped and stained with Coomassie Blue R-250 for verification of the loading sample.

### Quantitative RT-PCR Analysis

Quantitative RT-PCR was performed to characterize the expression profile of human target genes by using the human quantitative (q) RT-PCR arrays (Origene) per the manufacturer's instructions. Polymerase chain reaction was performed in 96-well optical plates using the iCycler (Bio-Rad Laboratories, Hercules, CA, USA) with primers specific for Prx I-VI, Trx1, Trx2, β-actin, glyceraldehyde 3-phosphate dehydrogenase (GAPDH), and iQ SYBR Green Supermix (Bio-Rad). The resulting fluorescence proportional to the amount of amplified DNA was measured at the end of each elongation phase at 530 nm. A standard graph of C_T _(the point at which the fluorescence crosses the threshold) values obtained from serially diluted target genes was constructed for all reactions to ensure that they were amplified and reported in proportion to template. C_T _values were converted to gene copy number of the template cDNA using the equation 2^ΔΔCT^. The ΔC_T _is the abundance of cDNAs for transcripts of each gene normalized to the β-actin and GAPDH at each time point. The ΔΔC_T _is obtained by subtracting a calibrator value for each gene transcript being assayed. In parallel with each cDNA sample, standard curves were generated to correlate C_T _values using serial dilutions of the target gene. The quality of the standard curve was judged from the slope and the correlation coefficient. Quantification was performed by comparing the fluorescence of a PCR product of unknown concentration with the fluorescence of several dilutions. Melting curve analysis was used for product validation. The primers for β-actin and GAPDH were supplied by Origene. Other primer sequences are summarized in Table 2.

**Table 2:** Sequence of Primers for Real-Time PCR^1 ^Amplification

**Primer for**	**Direction**	**Primer Sequence (5' to 3')**
**Human Prx I**	*Forward*	tttggtatcagacccgaagc
	
	*Reverse*	tccccatgtttgtcagtgaa

**Human Prx II**	*Forward*	ccagacgcttgtctgaggat
	
	*Reverse*	acgttgggcttaatcgtgtc

**Human Prx III**	*Forward*	gttgtcgcagtctcagtgga
	
	*Reverse*	gacgctcaaatgcttgatga

**Human Prx IV**	*Forward*	cagctgtgatcgatggagaa
	
	*Reverse*	taatccaggccaaatgggta

**Human Prx V**	*Forward*	ccctggatgttccaagacac
	
	*Reverse*	aagatggacaccagcgaatc

**Human Prx IV**	*Forward*	cgtgtggtgtttgtttttgg
	
	*Reverse*	tcttcttcagggatggttgg

**Human Trx1**	*Forward*	ctgcttttcaggaagccttg
	
	*Reverse*	tgttggcatgcatttgactt

**Human Trx2**	*Forward*	agcccggacaatatacacca
	
	*Reverse*	aatatccaccttggccatca

^1 ^Abbreviations: PCR, polymerase chain reaction; Prx, peroxiredoxin; Trx, thioredoxin.

### Statistical Analysis

Continuous data were reported with mean and standard error (S.E.M); ordinal data, with numbers and percentages. One-way analysis of variance (ANOVA) with Dunnett multiple comparison test and *t *test were performed using GraphPad Prism version 5.00 for Windows (GraphPad Software, San Diego, CA, USA). The summary *P *value is represented as a number of an asterisk. The test for linear trend between means and column numbers was used to investigate the linear trend of data set. Values were considered statistically significant if *P *< .05. In addition, Bonferroni multiple comparison was also performed. In this test, the value was considered statistically significant if *P *< .1.

## Results

### Preferential Increases of Prx I and Trx1 mRNA Expression as the Predominant Isoforms in Human Breast Cancer Tissue

**Figure 1:**
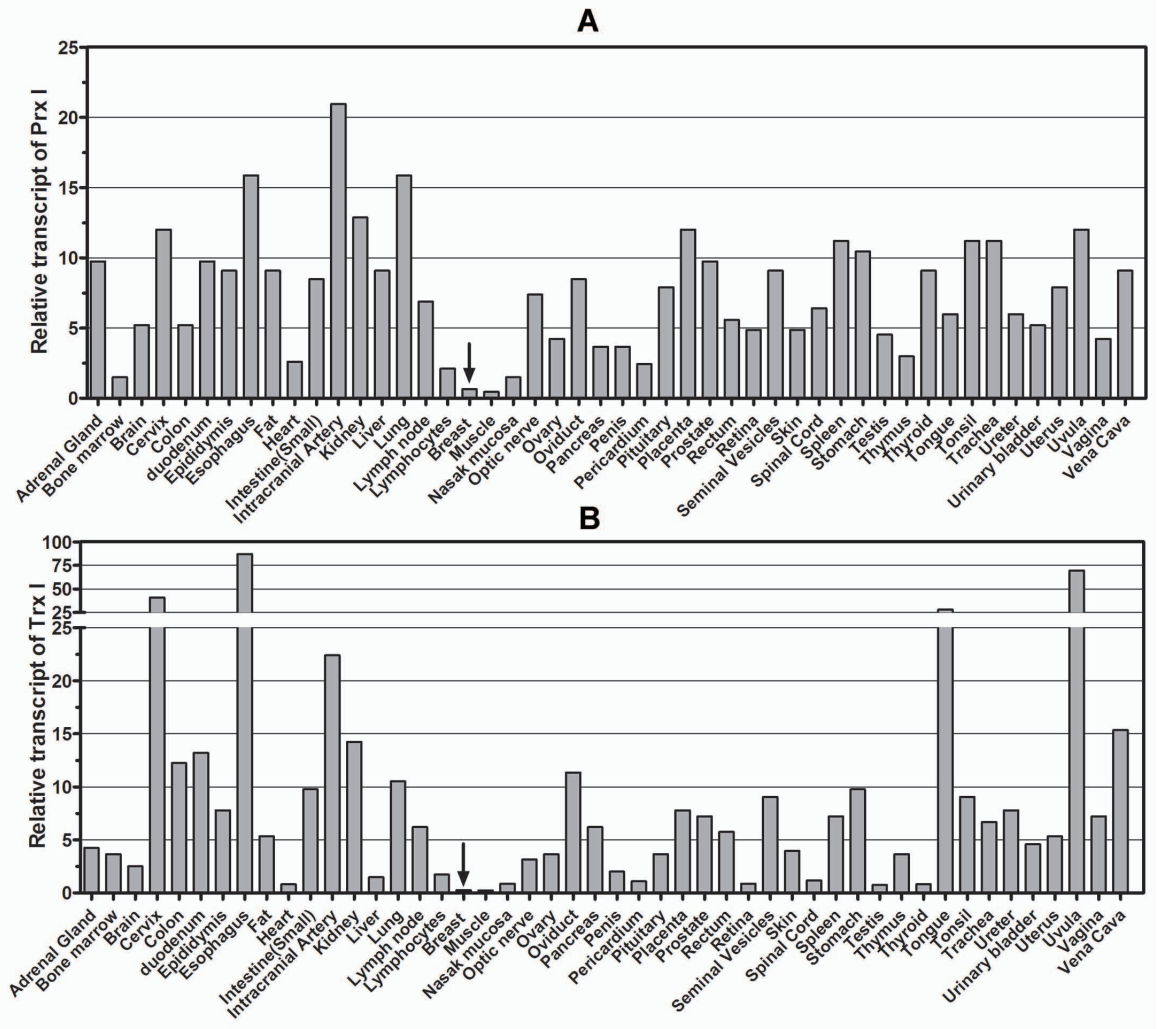
**Expression Profiles of Peroxiredoxin I and Thioredoxin1 in 48 Major Human Tissues**. The Human Major Tissue qRT-PCR array was used to determine transcript levels of Prx I (Figure 1A) and Trx1 (Figure 1B). For the human tissue array, tissues were selected from 48 individuals of different ethnicity. The y-axis represents the value of pg × 10^4 ^of DNA determined. Data were abtained using the comparative C_T _method with the values normalized to GAPDH levels and a standard curve. Details are in the "Materials and Method" section. Abbreviations: GAPDH, glyceraldehyde 3-phosphate dehydrogenase; Prx I, peroxiredoxin I; qRT-PCR, quantitative real-time polymerase chain reaction; Trx1, thioredoxin 1.

**Figure 2:**
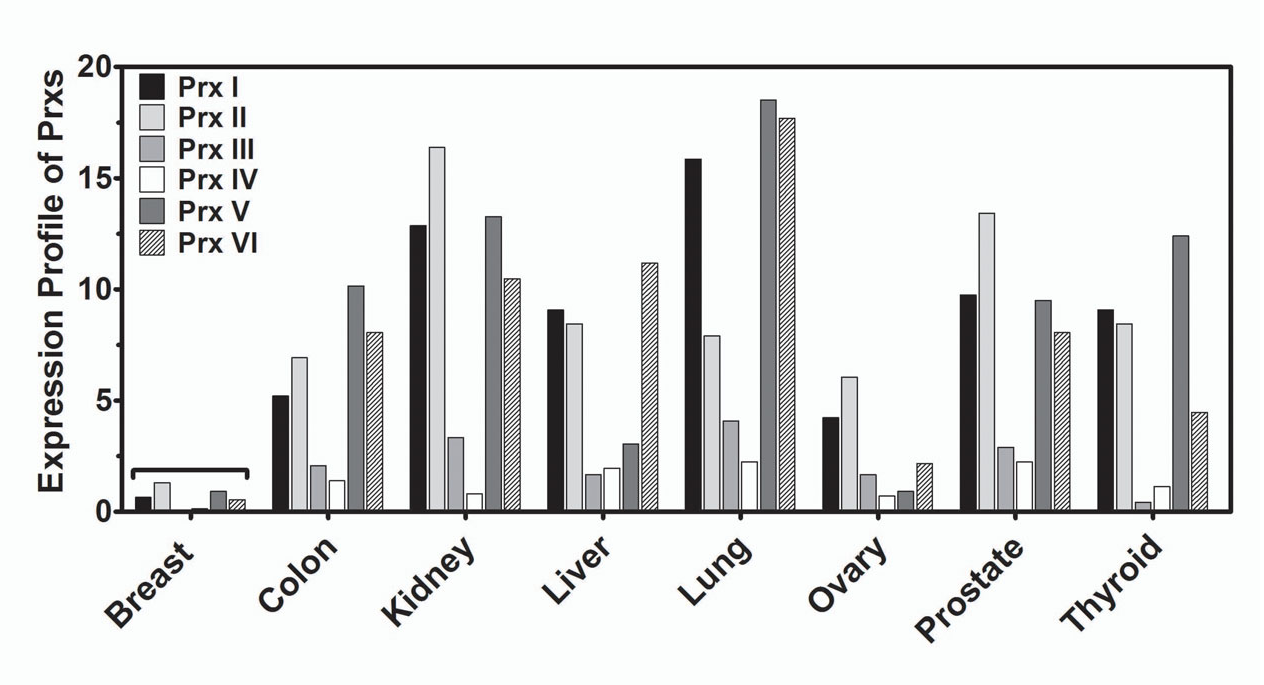
**Expression Profiles of Six Isoforms in the Peroxiredoxin Family in Major Human Tissues**. The Human Major Tissue qRT-PCR array was used to determine transcript levels of Prx I-VI. Expression profiles of 26 tissues are displayed. The profiles of the 40 other tissues were deleted in this figure to simplify the display. Other details are in the legend of Figure 1. Abbreviations: Prx, peroxiredoxin; qRT-PCR, quantitative real-time polymerase chain reaction.

**Figure 3:**
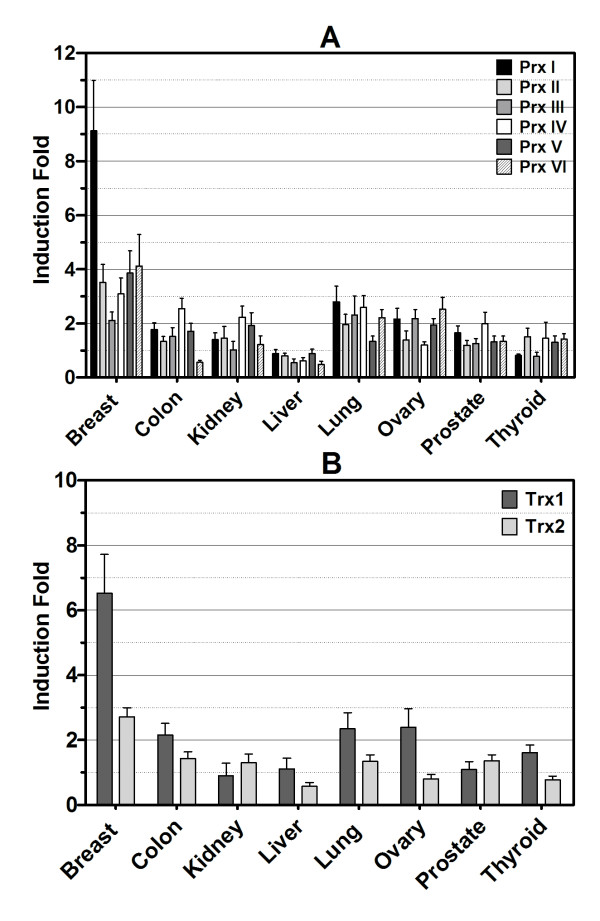
**Increased mRNA Levels of Peroxiredoxin and Thioredoxin Families in Eight Cancer Tissues Compared with Normal Tissues**. Cancer Survey qRT-PCR array was used to determine the transcript levels of Prx I-VI, Trx1, and Trx2 in breast, colon, kidney, liver, lung, ovary, prostate, and thyroid cancers. Samples in each of the eight cancer groups in the set of arrays consisted of three samples of normal tissue and nine samples of cancer tissues (cancer, phases I-IV) from different individuals. Data were analyzed using the comparative C_T _method with the values normalized to GAPDH levels. The y-axis represents the increase in the induction fold of the mRNA level of cancer tissue compared with the data from three samples of normal tissue. Error bar displays the range of standard error. Figure in *inset *is a scatter plot with individual values of the induction fold for Prx I depicted by each dot, the mean induction fold depicted by the longer horizontal line, and standard error depicted by the error bars (shorter horizontal lines) above and below the mean line. Clinicopathological information for each patient was provided by the supplier. Abbreviations: GAPDH, glyceraldehyde 3-phosphate dehydrogenase; mRNA, messenger RNA; Prx, peroxiredoxin; qRT-PCR, quantitative real-time polymerase chain reaction; Trx, thioredoxin.

Transcript levels of Prx I in breast tissue were very low (0.65 × 10^-4 ^pg), comparable to those in muscle (0.58 × 10^-4 ^pg), in which the Prx I level was lowest among 48 major human tissues (Figure 1A). Thioredoxin 1, as cytoplasmic electron donor to Prx I, was also expressed at the lowest level (0.24 × 10^-4 ^pg) among 48 major human tissues (Figure 1B). To address whether this low expression was specific to Prx I, we investigated mRNA levels of all members of the Prx family (Prx I-VI) using the same 96-well HMRT array. Expression profiles of each gene, shown in Figure 2, revealed that all levels of Prx were lowest in breast tissue when compared to the level of Prx in other tissues. The expression profiles of the Prx and Trx families in eight solid cancers (breast, colon, kidney, liver, lung, ovary, prostate, and thyroid) were studied using the CSRT 96-I array in which 12 samples (*n *= 3 for normal, *n *= 9 for corresponding cancer) from different individuals were included for each type of cancer for a total of 96 samples. As indicated in Figure 3A, the level of Prx1 mRNA was elevated in breast cancer by the highest fold (9.12 ± 1.86) among the eight types of solid tissue cancers, and the induction levels of Prx II-VI in breast cancer ranging from ~2- to ~4-fold) were not significantly different from those in other types of cancers (ranging from ~1- to ~3-fold). Figure 3B showed that Trx1 was also expressed at the highest level in breast cancer (6.47 ± 1.22), whereas Trx2 was not preferentially expressed in breast cancer (2.72 ± 0.28) (P = 0.0067).

**Figure 4:**
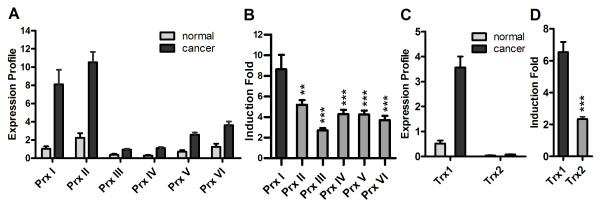
**Predominant Expressions of Peroxiredoxin I and Thioredoxin1 mRNA in Breast Cancer Tissue**. Data in Figure 4A and Figure 4C show the transcript levels of Prx I and Trx1, respectively. The BCRT II array (qRT-PCR) was used to determine the transcript levels of Prx I-VI, Trx1, and Trx2. Data were analyzed using the comparative C_T _method with the values normalized to β-actin level and expressed relative to controls. In parallel with each cDNA sample, standard curves were generated to correlate C_T _values using serial dilutions of the target gene. The y-axis represents the value of pg of DNA × 10^4^. The induction fold data shown in Figure 4B and Figure 4D were obtained from the expression profiles in Figure 4A and Figure 4C, respectively. The BCRT II array consisted of five samples of normal breast tissue and 43 samples of breast cancer tissues from different individuals. Clinicopathological information for each patient was provided by the supplier. Values are reported as mean ± standard error. The *t *test was performed for levels of induction fold for Prx I versus other Prx isoforms (Figure 4B), and for Trx1 versus Trx2 (Figure 4D). The *P *values are represented by asterisks (** = *P *< .01, *** = *P *< .001). Abbreviations: BCRT II, Human Breast Cancer qRT-PCR Array II; mRNA, messenger RNA; Prx, peroxiredoxin; qRT-PCR, quantitative real-time polymerase chain reaction; Trx, thioredoxin.

To examine the level of expression of Prx I and Trx1 among their families in breast cancer, we measured the expression levels for all members of the Prx and Trx families in breast cancer using a 48-well BCRT II array (Figure 4). In normal breast tissue, all Prx isoforms showed lower levels of expression compared with those of malignant tissues. Peroxiredoxin I and Prx II were predominant among the Prx isoforms as seen in Figure 4A (8.11 ± 1.58 × 10^-4 ^pg for Prx I, 10.53 ± 1.33 × 10^-4 ^pg for Prx II). Moreover, Prx II was expressed at the highest level in normal breast tissue among the isoforms (1.04 ± 0.23 × 10^-4 ^pg for Prx I, 2.25 ± 0.34 × 10^-4 ^pg for Prx II; P = 0.046 for Prx I *vs*. Prx II) (Figure 4A). In terms of induction fold of mRNA in breast cancer tissue, Prx I expression was highest among the six isoforms (8.64 ± 1.40 fold) (Figure 3B). For the Trx isoforms (Trx1 and Trx2), in both normal and malignant tissues, the expression level of Trx1 was much higher than that of Trx2 (Figure 4C). In Figure 4D, the higher-fold induction of Trx1 in malignant tissue is depicted compared with Trx2.

### Association of Prx I and Trx1 to Breast Cancer Grade

**Figure 5:**
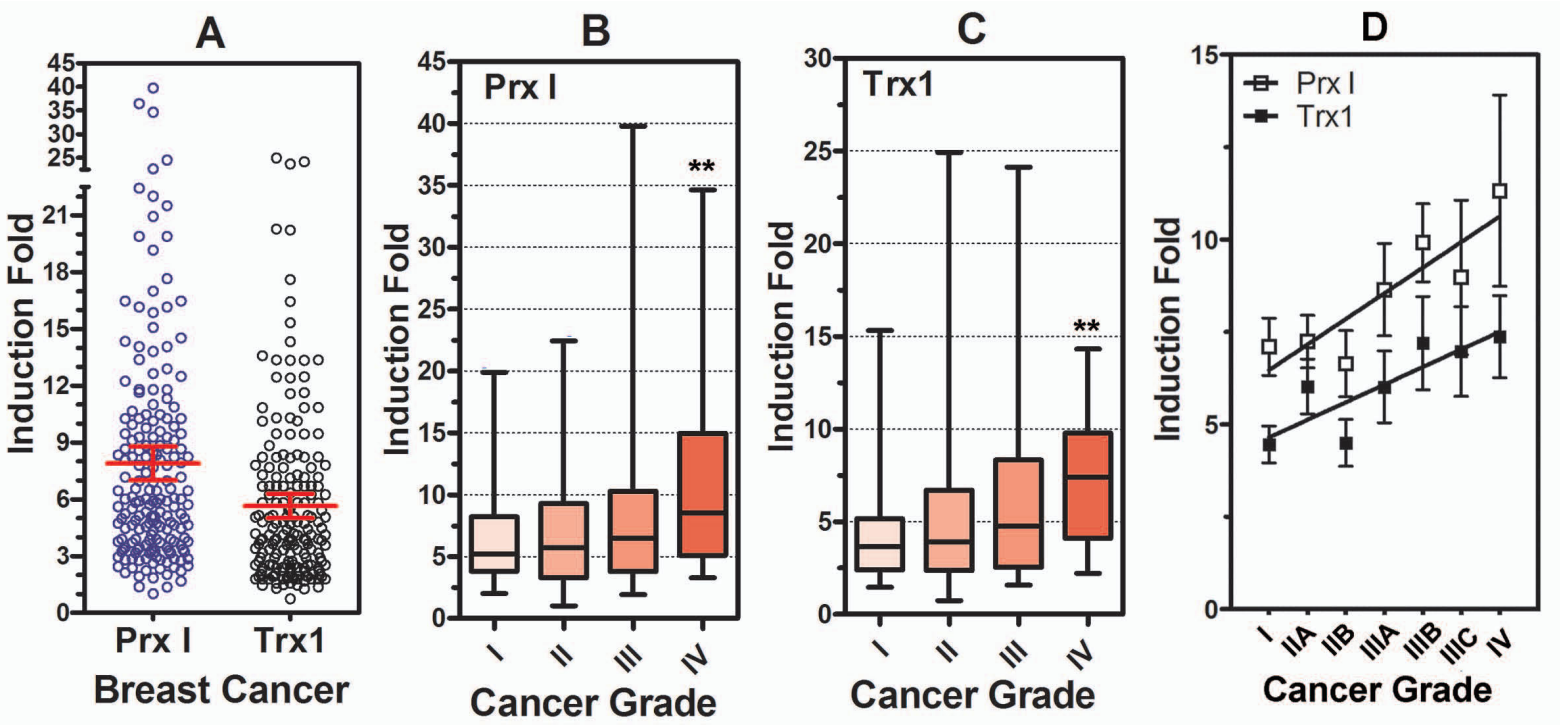
**Peroxiredoxin I and Thioredoxin1 mRNA Levels Associated with Grade of Breast Cancer**. Data from the breast cancer groups using the Cancer Survey qPCR array (*n *= 9) and Breast Cancer qRT-PCR array I-V (*n *= 176) are displayed as a scatter dot plot with mean and standard error (Figure 5A). Data for induction fold for each cancer grade are represented as box-and-whisker plots with minimum and maximum. The *t *test was performed to compare induction fold between grade I and grade IV (Prx I, Figure 5B; Trx1, Figure 5C). The *P *values are represented by asterisks (** = *P *< .01). In addition, the Bonferroni test for multiple comparison was also performed. In this test, the *P *value was considered statistically significant if *P *< .1. The number of samples per grade and subdivided grade was distributed as follows: grade I, 37; grade II, 76 (IIA, 44; IIB, 32); grade III, 60 (IIIA, 32; IIIB, 9; IIIC, 19); and grade IV, 12. All samples in grade IV (n = 12) represent metastatic cancer. To investigate the association between induction fold and cancer grade, one-way ANOVA test for linear trend was performed between mean induction fold and subdivided cancer grades (Figure 5D). For Prx I, slope = 0.6217, *P *= .02; for Trx1, slope = 0.4497, *P *= .02. For both cases, linear trends were considered statistically significant if *P *< .05. Clinicopathological information for each patient was provided by the supplier. Abbreviations: ANOVA, analysis of variance; Prx I, peroxiredoxin I; qRT-PCR, quantitative real-time polymerase chain reaction; Trx1, thioredoxin 1.

To evaluate the association of Prx I and Trx1 with grade of breast cancer, we measured mRNA levels in 204 samples of normal and malignant breast tissues ranging from 0 to IV grade by qRT-PCR and determined the induction fold from normal (grade 0) to malignant (grade I, II, III, IV). Expression of Prx I and Trx1 genes in breast cancer was assessed using five different sets of qRT-PCR arrays. Induction fold data were displayed as a scatter dot plot (Figure 5A). In breast cancer, 2-fold overexpression of Prx I occurred in 181 of 185 cases (97.8%), and 2-fold overexpression of Trx1 occurred in 168 of 185 cases (90.8%). Mean ± SEM induction folds were 7.90 ± 0.45 for Prx I and 5.64 ± 0.33 for Trx1.

To examine the relationship between mRNA expression of Prx I and Trx1 and progress of cancer, we displayed the data as box-and-whisker plots (cancer phase versus induction fold mRNA expression) (Prx I, Figure 5B; Trx1, Figure 5C). In both Prx I and Trx1, there was a significant relationship between the induction fold and increasing cancer phase, especially for metastatic cancer (comparison of Prx I expression from stage I to stage IV, *P *= .040; Trx1, *P *= .009). Stage IV (*n *= 12) was classified as metastatic cancer. In addition, we divided the cancer phases into subdivisions (stages I, IIA, IIB, IIIA, IIIB, IIIC, and IV) and compared these by induction fold expression. As shown in Figure 5D, induction fold was associated with subdivisions of cancer stages (*P *= .0181 for Prx I and P = .0191 for Trx1)

### Correlation Between Prx I and Trx1 in Human Breast Cancer

**Figure 6:**
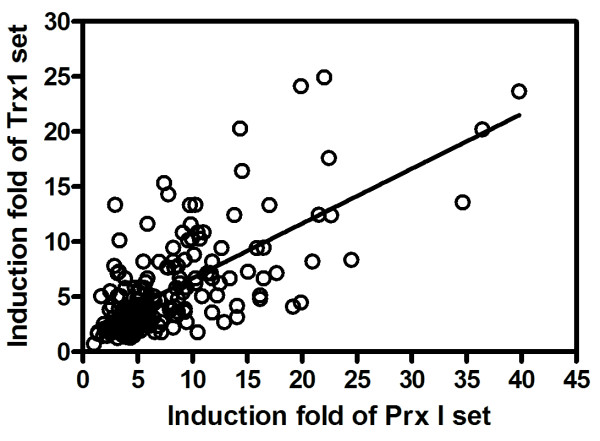
**Correlation Between Peroxiredoxin I and Thioredoxin1 mRNA Expressions in Breast Cancer**. Data of induction folds of Prx I and Trx1 in breast cancer shown in Figure 5A are displayed as a scatter plot. Details are in the legend of Figure 5. Abbreviations: Prx I, peroxiredoxin I; Trx1, thioredoxin 1.

To investigate an association between Prx I and Trx1 in human breast cancer, we plotted the both induction folds in breast cancer as x-y plot (x-axis for that of Prx I mRNA; y-axis for that of Trx1 mRNA). Figure 6 depicts the correlation between induction folds of Prx I and Trx1 genes in breast cancer (Pearson *r *= 0.6875; *P *< .0001), indicating an association between Prx I and Trx1 in breast cancer.

### Preferential Overexpression of Prx I and Trx1 Protein in Human Breast Cancer Tissue

**Figure 7:**
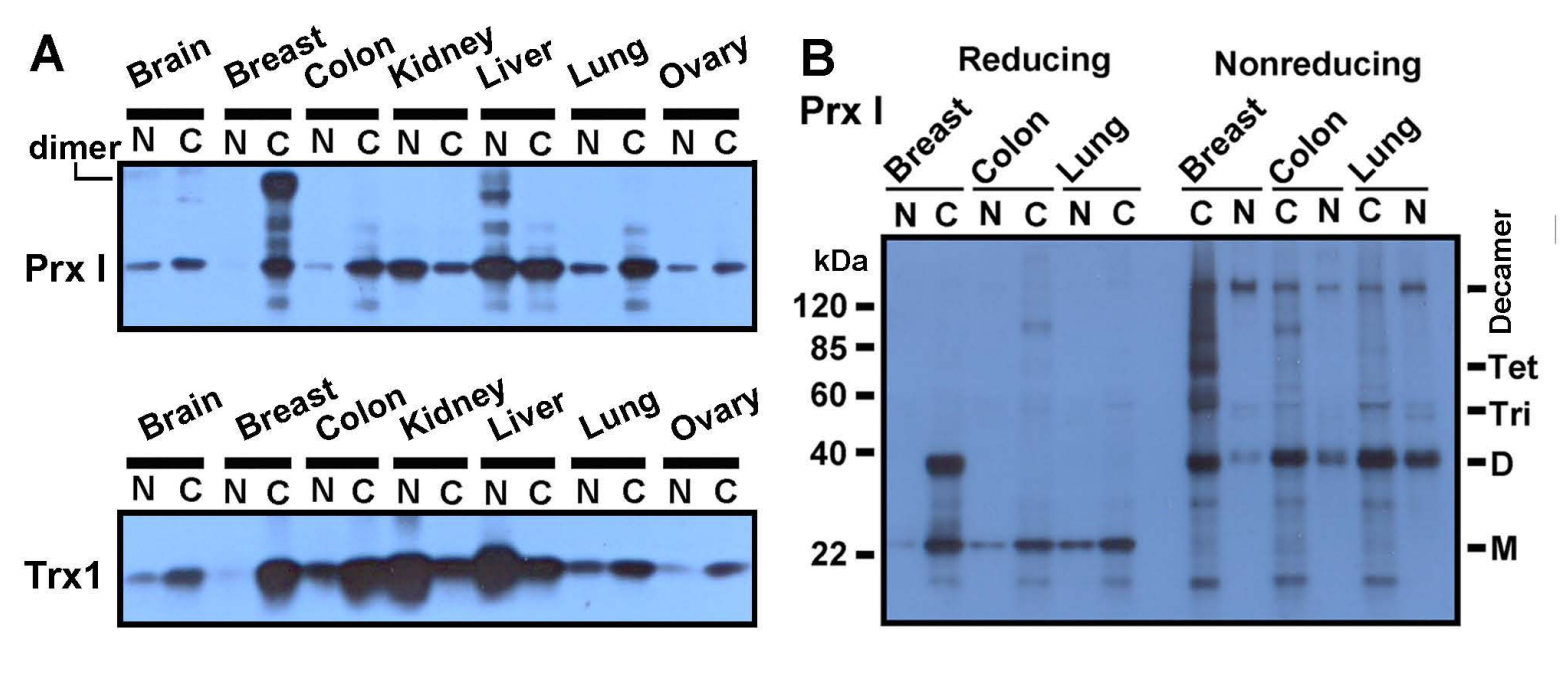
**Western Analysis of Peroxiredoxin I and Thioredoxin1 Protein Expressions in Malignant and Normal Tissues**. The total membrane and soluble protein lysates (15 μg) were loaded into reducing (Figure 7A and left side of Figure 7B) and nonreducing SDS-PAGE (right side of Figure 7B) and analyzed for protein expression. The sample information is described in Table 1. For example, N and C under the heading "Brain" are represented as BRN0 and BRC0 in Table 1, respectively. Figure 7B shows oligomerization for Prx I. Abbreviations: C, cancer (malignant); D, dimer; kDa, kilodalton; M, monomer; N, normal; Prx I, peroxiredoxin I; SDS-PAGE, sodium dodecyl sulfate polyacrylamide gel; Tet, tetramer; Tri, trimer; Trx1, thioredoxin 1.

To examine the expression of Prx I and Trx1 proteins, Western blot analysis was conducted of protein lysates from seven cancer tissue types (brain, breast, colon, kidney, liver, lung, and ovary) separated by SDS-PAGE. Both Prx I and Trx1 proteins appeared to be elevated at the highest level when compared with those of other tissues (Figure 7A). Western blot analysis of the human breast cancer samples revealed a band at approximately 40 kDa. Western blot analysis in Figure 7B showed that the band in the reducing gel was entirely shifted to several higher molecular weight forms as shown in the nonreducing gel, suggesting that the 40-kDa band represents the dimer form of Prx I. The presence of multimeric forms of Prx I, including the dimer and decamer forms, has been previously reported [29].

**Figure 8:**
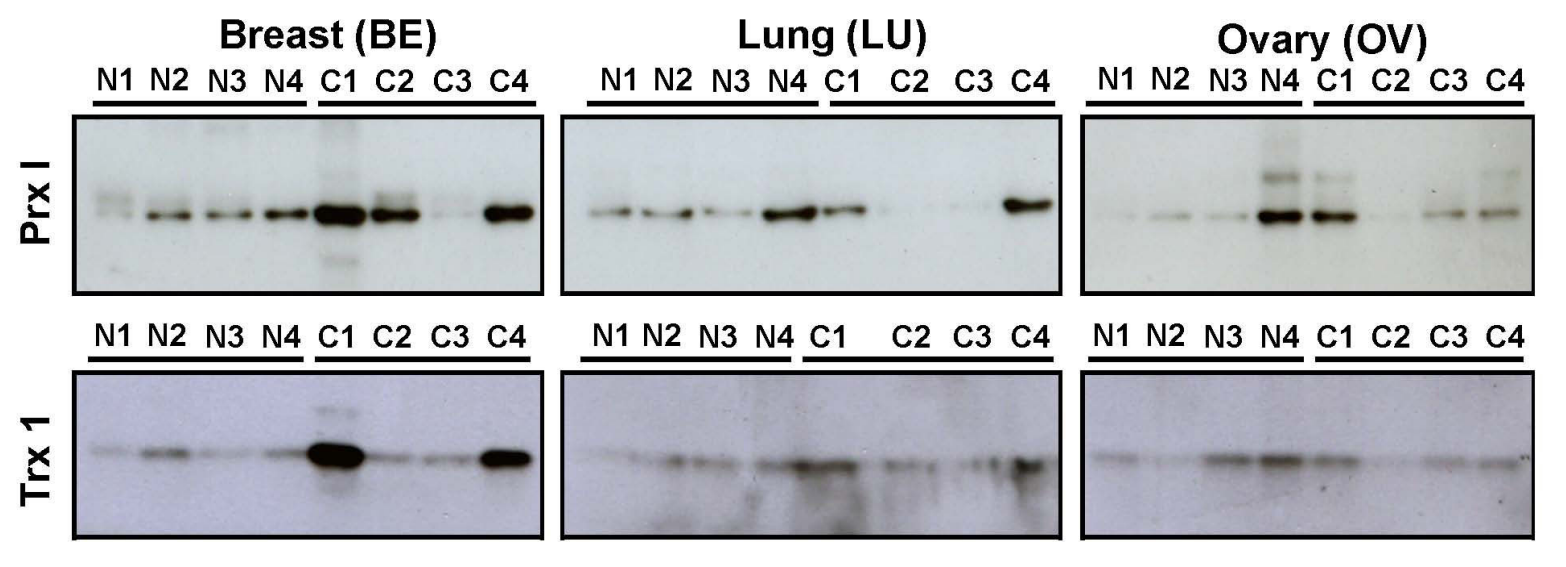
**Western Analysis of Peroxiredoxin I and Thioredoxin1 Protein Expressions in Malignant and Normal Tissues**. Four samples each of normal and cancer tissue providing total membrane and soluble protein lysates (15 μg) were loaded into reducing SDS-PAGE (right side of Figure 8B) and analyzed for protein expression. The sets of three blots with one antibody (breast [BE], lung [LU], and ovary [OV]) were exposed on the same film at the same time. The sample information is described in Table 1. For example, N1 and C1 under the heading of "Breast (BE)" are represented as BEN1 and BEC1 in Table 1, respectively. Abbreviations: C, cancer (malignant); N, normal; Prx I, peroxiredoxin I; SDS-PAGE, sodium dodecyl sulfate polyacrylamide gel; Trx1, thioredoxin 1.

Figure 8 displays Western blots for samples of four normal tissues and four cancer tissues from different individuals (different from the samples used in the previous experiment; see Table 1). The stronger band intensities for Prx I and Trx1 proteins indicate overexpression in breast cancer tissue, compared with those of lung and ovary.

**Figure 9:**
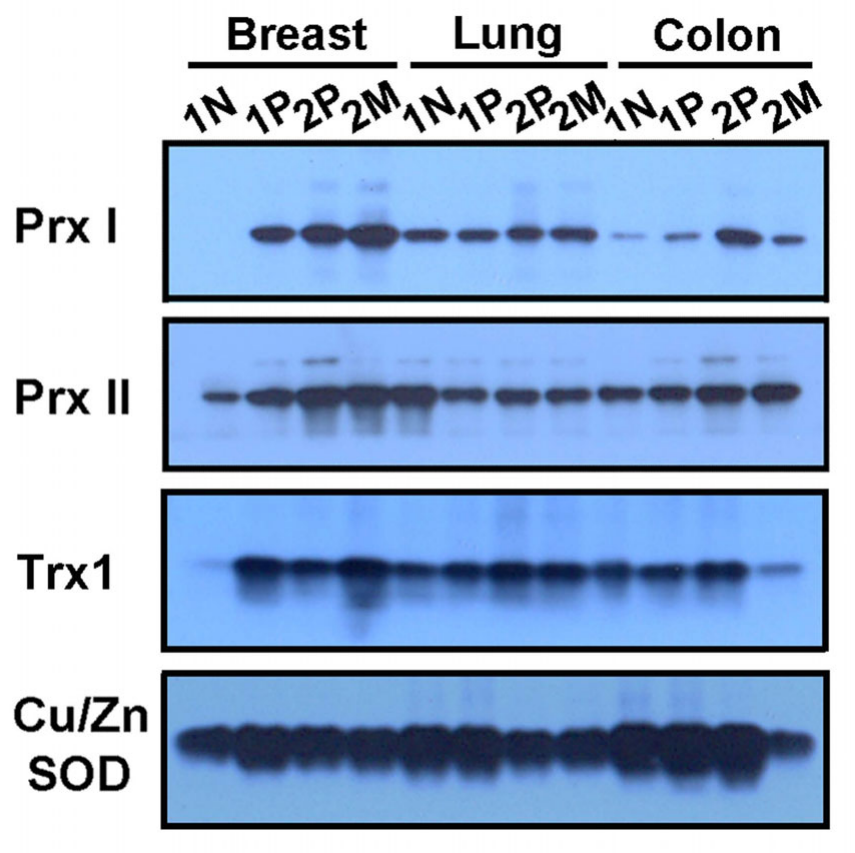
**Western Analysis of Peroxiredoxin I, Peroxiredoxin II, Thioredoxin1, and Copper/Zinc Superoxide Dismutase Protein Expressions in Paired Samples of Malignant and Distant Normal Tissue Homogenates of the Same Patient**. The total membrane and soluble protein lysates (15 μg) were loaded into reducing SDS-PAGE analyzed for protein expression. The patient information is described in Table 1. Cu/Zn SOD was included in this experiment as a positive control. Abbreviations: Cu/Zn SOD, copper/zinc superoxide dismutase; M, metastatic cancer; N, normal; P, primary cancer; Prx I, peroxiredoxin I; Prx II, peroxiredoxin II; SDS-PAGE, sodium dodecyl sulfate polyacrylamide gel; Trx1, thioredoxin 1.

A comparative Western blot analysis between the paired sets of breast tissue (paired normal and primary cancer from the same individual; paired primary and metastatic cancer from the same individual) and the paired sets of other tissues (lung and colon) revealed preferential overexpression of Prx I and Trx1 proteins in breast cancer compared with those in lung and colon cancer, and higher protein levels of Prx I and Trx1 in metastatic breast cancer than in primary breast cancer (Figure 9). Similarly, Prx II protein was overexpressed in breast cancer, but the Prx II protein level in normal tissue was significantly higher than that of Prx I in normal tissue. These comparative protein levels in normal and malignant tissues correspond with the levels of Prx II mRNA shown in Figure 4A.

These Western data shown in Figures 7, 8, 9 indicate that Prx I protein was overexpressed in 7 of 8 cases (87.5%) of breast cancer but in none of the 6 cases of normal breast tissue. Thioredoxin1 protein was overexpressed in 6 of 8 cases (75.0%) of breast cancer.

## Discussion

To our knowledge, there has been only one previous report suggesting overexpression of Prx I protein in human breast cancer. Overexpression of Prx I was detected in 21 of 24 patients (87.5%) with breast cancer, but no significant relationship was found between overexpression of Prx I and progress in breast cancer [13]. Their finding of overexpression of Prx I protein in breast cancer tissue by Western immunoblotting agrees with our observations (7 of 8 cases, 87.5%; 0 of 6 normal, 0%). One study has examined the association of overexpression of Prx I protein with clinicopathological parameters in oral cancer [15]. Low Prx I expression in oral cancer was associated with larger tumor mass and poorly differentiated cancer cells. In our study, all samples of breast cancer stage IV, which belonged to metastatic breast cancers, were found to overexpress Prx I at the highest level. Moreover, in our study of 204 samples, Prx I expression was significantly associated with increasing cancer progress.

We examined all six members of the Prx family in eight human cancers (breast, colon, kidney, liver, lung, ovary, prostate, and thyroid) and found that Prx I was preferentially induced only in breast cancer, not in other cancer tissues. The isoforms Prx I and II were highly expressed in breast cancer. The expression level of Prx II was slightly higher than that of Prx I in breast cancer, but the induction fold of Prx I was significantly higher than that of Prx II. This apparent inconsistency seems to be caused by the lower level of Prx I mRNA in normal breast tissue compared with that of Prx II.

At present, few studies have been conducted on all six Prx members in various human cancers [13,16]. In contrast to our observations, other results have shown high protein expression of Prxs III, IV, and V in breast cancer, but not Prxs I, II, V, and VI. Immunoreactive protein and mRNA levels do not necessarily correspond with each other, as previously seen in a study of Prx V in rat tissues [30]. This suggests that both translational and posttranslational mechanisms probably have effects on Prx protein expression in human tissues. For example, destabilizing Prx proteins by overoxidation or phosphorylation leads to degradation, which results in reduced protein levels in cancer tissue [31,32]. Such posttranslational modification in cancer tissue could be one of the reasons for the discrepancy between mRNA and protein levels of Prx I in breast tissue. A more reliable approach may be to assess the level of transcription in cancer samples. In our study, we attempted to measure transcription.

As an electron donor, Trx is essential to cellular function of Prx [17]. Of the two isoforms of human Trx, cytoplasmic Trx1 and mitochondrial Trx2, we focused on Trx1 in breast cancer tissue because of its localization with Prx I. As with Prx I, there are many reports that Trx1 may be closely associated with cancers. Previous studies have shown the expression of Trx1 in a number of human cancer tissues [26-28,33,34]. Estrogen receptors and p53 are important transcription factors in the growth regulation of cancer cells in breast cancer. Previous findings suggest that Trx1 expression is linked to the estrogen receptor-dependent and p53-dependent regulation of growth in breast cancer cells [34]. This observation is consistent with the association of Trx1 and breast cancer. In the present study, we demonstrated that increasing Trx1 expression was associated with progress of breast cancer, and that Trx1 was correlated with Prx I in human breast cancer. It is not certain that, in breast cancer, the involvement of Trx1 reflects its ability to regulate Prx I action, but at least in terms of magnitude of expression in the same patients, the association of Trx1 with Prx1 supports the theory that their functions are related to each other in breast cancer.

## Conclusion

We have demonstrated here that Prx I and Trx1 are preferentially overexpressed in human breast carcinoma and the expression levels are associated with tumor grade. In addition, Prx I expression is correlated with Trx1 expression in breast cancer. We found that mRNA levels for both Prx I and Trx1 in normal breast tissue are extremely low compared with those of other major human tissues. Based on these observations, we suggest that Prx I and Trx1 could be used as potential breast cancer markers.

## Abbreviations

ROS: reactive oxygen species; Prx: peroxiredoxin; Trx: thioredoxin; DTT: dithiothreitol; SDS: sodium dodecyl sulfate; PAGE: polyacrylamide gel electrophoresis; PCR: Polymerase chain reaction.

## Competing interests

The authors declare that they have no competing interests.

## Authors' contributions

IHK conducted the work, analyzed the data and wrote the manuscript. MKC and KHS performed the experiments throughout this work. All authors have read and approved the final manuscript.
